# Communication Between Epithelial–Mesenchymal Plasticity and Cancer Stem Cells: New Insights Into Cancer Progression

**DOI:** 10.3389/fonc.2021.617597

**Published:** 2021-04-21

**Authors:** Xiaobo Zheng, Fuzhen Dai, Lei Feng, Hong Zou, Li Feng, Mingqing Xu

**Affiliations:** ^1^Department of Liver Surgery, West China Hospital, Sichuan University, Chengdu, China; ^2^Department of General Surgery, The First People's Hospital of Longquanyi District, Chengdu, China; ^3^Department of Biliary Surgery, West China Hospital, Sichuan University, Chengdu, China; ^4^General Surgery Center of PLA, General Hospital of Western Theater Command, Chengdu, China; ^5^Department of General Surgery, Hospital of Chengdu University of Traditional Chinese Medicine, Chengdu, China; ^6^Department of Hepatopancreatobiliary Surgery, Meishan City People's Hospital, Meishan Hospital of West China Hospital, Sichuan University, Meishan, China

**Keywords:** epithelial-mesenchymal plasticity, cancer stem cells, epithelial-mesenchymal transition, mesenchymal-epithelial transition, metastasis, stemness

## Abstract

The epithelial–mesenchymal transition (EMT) is closely associated with the acquisition of aggressive traits by carcinoma cells and is considered responsible for metastasis, relapse, and chemoresistance. Molecular links between the EMT and cancer stem cells (CSCs) have indicated that EMT processes play important roles in the expression of CSC-like properties. It is generally thought that EMT-related transcription factors (EMT-TFs) need to be downregulated to confer an epithelial phenotype to mesenchymal cells and increase cell proliferation, thereby promoting metastasis formation. However, the genetic and epigenetic mechanisms that regulate EMT and CSC activation are contradictory. Emerging evidence suggests that EMT need not be a binary model and instead a hybrid epithelial/mesenchymal state. This dynamic process correlates with epithelial–mesenchymal plasticity, which indicates a contradictory role of EMT during cancer progression. Recent studies have linked the epithelial–mesenchymal plasticity and stem cell-like traits, providing new insights into the conflicting relationship between EMT and CSCs. In this review, we examine the current knowledge about the interplay between epithelial–mesenchymal plasticity and CSCs in cancer biology and evaluate the controversies and future perspectives. Understanding the biology of epithelial–mesenchymal plasticity and CSCs and their implications in therapeutic treatment may provide new opportunities for targeted intervention.

## Introduction

Epithelial–mesenchymal transition (EMT) is the process through which epithelial cells alter their phenotype, enabling them to lose their main epithelial cell traits and convert into cells expressing mesenchymal cell markers (1, 2). Following the EMT, cells switch from polygonal to spindle-like fusiform shape, lose cell polarity, and gain increased resistance to apoptosis and the ability to migrate and invade (3–8). The EMT occurs in various physiological and pathological conditions, including embryonic processes essential for normal development, tissue morphogenesis and repair, tissue reconstruction, fibrogenesis, and tumorigenesis (9–12).

EMT has been associated with cancer stemness, characterized by an increase in the number of cancer stem cells (CSCs) (13–16). CSCs are subclone cells in the tumor tissue that obtain stem cell traits (17, 18). CSCs exhibit self-renewal, maintain tumor formation ability, and can differentiate into various cells to support the tumor; therefore, these cells are considered a source of tumorigenesis, metastasis, and relapse (19–21). In addition, CSCs are highly related to chemoresistance (22). Even if most tumor cells are eliminated by chemotherapy, if CSCs are not eradicated, relapse, metastasis, and chemoresistance still commonly occur (23). Similar to normal stem cells, most CSCs are quiescent and slow growing, which is why they are resistant to anticancer drugs (24).

Previous studies have suggested that cells undergoing EMT possess stem cell traits and that tumor cells retaining stemness express markers of the EMT (25). However, several studies have shown that stemness is coupled with the mesenchymal–epithelial transition (MET) rather than the EMT and that regulation of the EMT and stemness is distinct (26–30). Thus, the correlation between the EMT and stemness is not clear. Recently, the EMT was defined as a dynamic, hybrid epithelial/mesenchymal state (31–33). Of note, this hybrid state is a coexistence of epithelial and mesenchymal phenotypes, rather than the junction of separate phenotypes; epithelial–mesenchymal plasticity was used to describe this hybrid epithelial/mesenchymal state during EMT. Importantly, epithelial–mesenchymal plasticity is involved in cancer progression and associated with stem cell-like traits, which may help explain the conflicting relationship between EMT and CSCs (34, 35). In this review, we discuss the relationship between EMT/MET/epithelial–mesenchymal plasticity and CSCs.

## EMT Confers Tumor Cells With Traits of CSCS

Stemness acquired after the induction of the EMT provides cells with the traits of increased migratory ability and antitumor drug resistance and promotes tumor metastasis and recurrence (36–38). Dang et al. (39) found that the EMT induced by transforming growth factor (TGF) correlates with acquisition of tumor-initiating stem cells (TISCs) in breast cancer. SNAIL directly regulates the expression of Nanog in mesenchymal cells generated by the EMT. Deletion of SNAIL influences the growth but not the initial formation of the tumor. Kim et al. (40) found that CD13^+^ liver CSCs can survive in a hypoxic environment after chemotherapy and that EMT enhances cell stemness by suppressing the activity of reactive oxygen species. Garg suggested that CSCs could be classified into two distinct functional transition states, one of which is cyclic CSCs with predominant epithelial phenotype that can self-renew and differentiate into mature cancer cells. The other subset is autophagic/non-cyclic CSCs with predominant mesenchymal phenotype that have the capacity to invade and metastasize and that are majorly responsible for cancer mortality (41). The EMT seems to work together with the microenvironment to promote proliferation and homing of CSCs. Cytokines are critical for regulating the microenvironment and are necessary for initiating the EMT (42). It was reported that the TGF-β/BMP signaling pathways regulate primary tumor and metastasis microenvironments in colorectal and breast cancer (43). Collectively, these studies point to the fact that CSCs can be induced by EMT and exhibit a mesenchymal phenotype with greater metastatic potential.

EMT and acquisition of CSC-like characteristics are key steps in the metastasis and recurrence of a tumor after radical resection. A study by Mani and colleagues (25) showed that EMT induction in immortalized human mammary epithelial cells led to increased mammosphere formation *in vitro* and tumorigenicity *in vivo*, suggesting that the EMT process can stimulate the acquisition of cell stemness. Moreover, the EMT confers stem cell-like properties, consistent with the migratory CSC concept. Several lines of evidence have supported the relationship between EMT and stemness. For example, Morel et al. (44) showed that CD44^−^CD24^+^ breast epithelial cells, which are non-tumorigenic, can induce the EMT after activating the RAS/mitogen-activated protein kinase pathway and acquire the CD44^+^CD24^−^ phenotype and stem cell traits. CD24^+^ cells treated with TGF do undergo the EMT, as demonstrated by the downregulation of E-cadherin and the upregulation of vimentin, accompanied by acquisition of CD24^−^ features. It is thus tempting to speculate that the EMT may be an important step in controlling the transition of CD44^−^CD24^+^ cells to CD44^+^CD24^−^ cells. In CSCs isolated from colorectal cancer surgery samples and analyzed using gene chips, Hwang et al. (45) found a high expression of CD44 and CD166, stem cell markers that regulate the EMT-associated transcription factor (TF) SNAIL. Subclones of basement-like breast cancer cells have a high proportion of CD44^+^CD24^−^ stem cell-like cells and overexpress EMT-correlated genes (46). Another study found that Fox2, an EMT-related TF, is highly expressed in breast cancer and is closely linked with basement-like subclones (47). Immunohistochemical analysis of 479 infiltrated breast cancer samples revealed that basement-like breast cancers express high levels of EMT-associated factors but low levels of E-cadherin (48). Taken together, these studies confirmed the fact that stemness and EMT are indeed intricately linked.

## MET in Recovering Epithelial Traits for Enhancing Stemness to Facilitate Distal Colonization

Several results have linked the MET and stem cell-like traits, challenging the view on the relationship between the EMT and CSCs (31, 49–51). The general view suggests that EMT-related transcription factors (EMT-TFs) must be downregulated in order to convert mesenchymal cells into epithelial cells and to increase proliferation, thereby promoting tumor metastasis formation (52). In order to form clones, malignant tumor cells need to assume an epithelial phenotype and maintain a state of stemness (53). Interestingly, Padmanaban et al. revealed that the expression of E-cadherin needs to be rescued *via* the inhibition of TGFβ-receptor signaling during the detachment, systemic dissemination, and seeding phases of metastasis in invasive breast ductal carcinomas (54). Fibroblasts must experience the MET to complete their progression to induced pluripotent stem cells (29). A study by Tsai et al. (27) clearly supported the role of the EMT in dissemination and the subsequent MET for colonization and macrometastasis. Additionally, a study by Ocaña et al. (28) also supported the role of the EMT in dissemination and the necessity of reversing the EMT for metastasis.

The mechanism underlying MET in recovering cancer cells stemness is still complicated. Several studies have reported that downregulation of traditional EMT-TFs cannot induce stemness because the regulation of stem cell properties is independent from that of epithelial plasticity (55). However, stem cell properties can be acquired through inhibition of Prrx1 (28, 56). Prrx1 and TWIST can both induce EMT properties alone; however, while deletion of Prrx1 induces lung metastasis, ablation of TWIST does not have the same effect. Intriguingly, deletion of Prrx1 in BT-549 cells enhances stemness, accompanied with increased mammosphere formation, self-renewal ability, and CD24^−^/CD44^+^ CSC proportion (28). Instead, downregulation of TWIST does not induce stemness, suggesting that downregulation of traditional EMT-TFs is not related to the occurrence of stemness and that regulation of the EMT and CSCs is distinct. In addition, Prrx1 expression predicts better prognosis and higher metastasis-free survival (57). Celià-Terrassa et al. (58) illustrated that the EMT can inhibit TISCs, suggesting that there are different subpopulations of EMT-TFs. In addition, the EMT must be reversed to allow growth and clonal expansion because invasive dedifferentiated tumor cells from the EMT were found to be quiescent, whereas proliferation was detected in redifferentiated metastatic tumor cells, suggesting that the EMT should reverse to the MET.

It has been reported that the mesenchymal state is related to early events in metastasis, such as dissemination, invasion, and intravascular infiltration. EMT-TFs initiate the invasion–metastasis cascade when aberrantly activated in tumors. A recent study showed that CSC formation is an early and frequent event in LSC progression (59). It has also been suggested that the epithelial state with stemness is correlated with later phases of metastasis (55). Moreover, the observations that CSC plasticity is elevated in advanced cancers and that regulation of the epithelial–mesenchymal states is increased are highly relevant (35). Mesenchymal state-associated invasion and dissemination are necessary, but not sufficient, to induce metastasis, and additional epithelial state with stemness is required to complete the full metastasis cascade (60). Therefore, epithelial–mesenchymal heterogeneity with stemness plasticity is involved in the entire process of invasion and metastasis.

## Emergence of Epithelial–Mesenchymal Plasticity

Recently, epithelial–mesenchymal plasticity was recommended as unified nomenclature by the EMT international association and was termed as the ability of cells to adopt mixed epithelial/mesenchymal (E/M) features and transit between EMT and MET states (50, 61). This epithelial–mesenchymal plasticity has been variably referred to as partial EMT, hybrid E/M status, intermediate EMT, a metastable EMT state, EMT continuum, and EMT spectrum, which were widely used in past studies (62). The cells undergoing epithelial–mesenchymal plasticity express a mixture of epithelial and mesenchymal features and express both epithelial and mesenchymal markers (63). Epithelial–mesenchymal plasticity also helps to account for the reversibility of the EMT process. Epithelial cells going through EMT give rise to cell populations that may enter reversibly into states with various proportions of epithelial and mesenchymal features (64, 65). Epithelial–mesenchymal plasticity is thought to provide cells with the fitness and flexibility to fulfill the diverse requirements during the course of either developmental or pathological processes. These cell transitions allow them to migrate from the primary tumor and invade the secondary site, playing a fundamental role in cancer metastasis. Epithelial–mesenchymal plasticity is associated with tumor cell migration, invasion, colonization, stemness, and drug resistance (66).

Epithelial–mesenchymal plasticity has been reported in many studies. The hybrid E/M phenotypes have been confirmed both *in vitro* and *in vivo*. Huang et al. systematically analyzed the protein levels of the epithelial and mesenchymal markers in 42 ovarian carcinoma cell lines. Among these 42 cell lines, 9 have been characterized as epithelial cells, 7 as mesenchymal cells, and the remaining 26 cell lines were characterized as hybrid E/M phenotypes (67). The existence of hybrid E/M states has also been observed in animal models. Pastushenko and colleagues screened a large panel of cell surface markers, such as EpCAM, vimentin, CD106, CD61, and CD51, in genetic mouse models of skin and mammary primary tumors. They identified the existence of multiple tumor subpopulations associated with different EMT stages: from completely epithelial to completely mesenchymal states, passing through intermediate hybrid states (68). The hybrid E/M phenotypes were also detected in clinical samples. Metastatic breast cancers were categorized as either having an epithelial or hybrid phenotype using a prediction algorithm, where the *VIM:CDH1* gene expression ratio was combined with the expression of *CLDN7* (69). A partial EMT process including the upregulation of mesenchymal genes in conjunction with the downregulation of certain epithelial genes was confirmed in a subset of HNSCC cells through single-cell RNA sequencing (70). Taken together, the epithelial–mesenchymal plasticity in cancer cells describes the presence of both epithelial and mesenchymal markers in the same cancer cells. It might reflect a stable state of cancer type or a transition phase of cancer cells while they are switching their phenotype. Its correlation with aggressiveness and metastasis further enforces the crucial role of epithelial–mesenchymal plasticity in cancer progression.

## The Interplay Between Epithelial–Mesenchymal Plasticity and CSCS

Distal tumor subclone formation is thought to be a multistep and long-term process, which also explains why various subclones have distinct proliferative abilities (71). Stemness is also a state of plasticity in tumor progression, allowing static and migratory CSCs to coexist (72). These ideas are consistent with the concept of epithelial–mesenchymal plasticity, a process that is thought to be a hybrid state during metastasis. The shift among the hybrid states of EMT may orchestrate the entire process of distant metastasis formation, from acquisition of invasive ability from primary tumor and dissemination *via* the bloodstream, to seeding in distant organs, stemness recovery for clonal expansion, and macrometastasis (73). Emerging evidence from theoretical and experimental studies has revealed the association of epithelial–mesenchymal plasticity with CSCs (74–77). Pastushenko et al. reported that the earliest EMT state already exhibits increased CSC frequency, and tumor stemness does not increase further in later hybrid epithelial–mesenchymal states (68). Francescangeli et al. reported that a pre-existing population of ZEB2^+^ quiescent cells in colorectal cancer showed both stemness and mesenchymal features and dictated chemotherapy resistance (78). Co-expression of stem cell and both epithelial and mesenchymal characters was also observed in circulating tumor cells of bladder cancer patients (79). Epithelial–mesenchymal plasticity was associated with miRNA let-7, which was an important factor affecting the CSC phenotype in high-grade serous ovarian carcinoma samples and could be correlated with tumor growth and metastasis (80). Quan et al. reported that ~60% of the leader CSCs in collective invasion co-existed with hybrid epithelial–mesenchymal states, indicating that CSCs with epithelial–mesenchymal plasticity play a key role in cancer cell collective invasion (81). Moreover, a previous study reported that in response to microenvironmental signals, lung cancer cells converted to CSC state through regulation of the balance between epithelial and mesenchymal transition (82). Collectively, the above results indicate that epithelial–mesenchymal plasticity confers cancer cells with the traits of stemness.

The mechanism underlying epithelial–mesenchymal plasticity and CSCs is still largely unknown. Recently, some factors that regulate the epithelial–mesenchymal plasticity and stemness of CSCs were reported. OvoL/Shavenbaby factors are a family of key epithelial stabilizers and are critical for adult stem cell homeostasis. Stemness and epithelial–mesenchymal plasticity could be regulated by interaction of EMT transcription factors and OvoL/Shavenbaby (83). A study reported that the non-coding RNAs expressed on the DLK1-DIO3 locus regulate the epithelial–mesenchymal plasticity in breast epithelial progenitor cells, providing evidence of the interplay of epithelial–mesenchymal plasticity and stemness (84). miRNAs, which are important factors in tumorigenesis and progression of cancers, are also involved in mediating interactions between epithelial–mesenchymal plasticity and CSCs (85–88). Jiang et al. reported that Prrx1 promotes epithelial–mesenchymal plasticity and activates cell dormancy in head and neck squamous cell carcinoma and that miR-642b-3p restoration rescues PRRX1-induced phenotype and cell dormancy (85). Furthermore, You et al. observed that miRNA-495 confers inhibitory effects on CSCs, as well as EMT, in oral squamous cell carcinoma through HOXC6-mediated TGF-β signaling pathway (86). The long non-coding RNA H19 mediates epithelial–mesenchymal plasticity by differentially sponging miR-200b/c and let-7b, wherein the latter is a CSC regulator in colon cancer (88, 89). Several studies have shown that cells with hybrid E/M states and CSC phenotypes are spatially segregated in the primary tumor (90). Bocci et al. observed through a mechanism-based dynamical model that the diffusion of EMT-inducing signals such as TGF-β, together with non-cell autonomous control of EMT and CSC decision-making *via* the Notch signaling pathway, can explain the experimentally observed disparate localization of subsets of CSCs with varying EMT phenotypes in the tumor (74). These results offer insights into the principles of spatiotemporal patterning in epithelial–mesenchymal plasticity and identify a relevant target during hybrid E/M states to alleviate multiple CSC subsets. Using a mechanism-based model, Bocci et al. explained how metformin can both inhibit EMT and blunt the aggressive potential of CSCs simultaneously by driving the cells out of a hybrid E/M stem-like state with enhanced Notch-Jagged signaling (91).

The expression levels of EMT-TFs, such as SNAIL and TWIST, in primary tumors are also fluctuant associated with cancer cell stemness. The consecutive expression of SNAIL, Prrx1, and TWIST also inhibits the formation of metastasis because EMT-TFs must be downregulated to facilitate stemness recovery and tumor formation (58). This is not contradictory, but simply reflects epithelial–mesenchymal plasticity and the dynamic process resembling the migration of embryonic cell populations to distant organs/sites. Thus, EMT-TFs are related to cell behavior rather than to cell fate, and hence, their expression is dynamic. Accordingly, as recommended by the EMT international association, EMT-TFs alone cannot be used as markers of differentiated cell populations, the equivalent of differentiated distant metastases (92). Further investigation of the downregulation of EMT-TFs in signaling pathways associated with the formation of CSCs is needed, particularly with regard to the interplay among the epithelial–mesenchymal plasticity, invasion of the primary tumor, and stemness recovery for tumor metastatic colonization. The complex interplay between epithelial–mesenchymal plasticity, CSCs, and tumor microenvironment gives rise to tumor heterogeneity that still represents the major challenge hampering therapy for metastasis and chemoresistance.

## Conclusion and Perspectives

This review suggests that epithelial–mesenchymal plasticity is involved in the process of CSC development. The coexistence of epithelial–mesenchymal plasticity and CSCs correlates with poor prognosis and resistance to therapy (93). Furthermore, emerging evidence has shown that targeting epithelial–mesenchymal plasticity-induced CSCs can effectively regulate tumor progression and drug resistance. Liu et al. reported that metformin inhibits prostate cancer resistant to enzalutamide by reducing the cells with hybrid E/M status and, thereby, restricting the formation of CSCs (94). Nevertheless, the mechanism underlying the relationship between epithelial–mesenchymal plasticity and CSCs still remains poorly understood. Additional research is required at the molecular level to clarify, for example, how TWIST and Prrx1 interact, and how Prrx1 inhibits the induction of stemness while not inhibiting the EMT-promoting function of TWIST, as well as to elucidate the roles of other potential EMT-related factors, such as SNAIL and ZEB1. This will help to unveil the mechanisms underlying CSC initiation and tumor metastasis. In addition, it is plausible that non-CSCs can transition to CSCs during the dynamic process of epithelial–mesenchymal plasticity. Therefore, the plasticity of CSCs needs to be considered to explore therapeutic strategies aimed at overcoming tumor heterogeneity and chemoresistance by targeting CSCs. In all, understanding these molecular mechanisms can help improve the efficiency of the ongoing and planned therapeutic trials to control cancer progression, treatment resistance, and disease recurrence.

## Author Contributions

MX proposed the research. XZ, FD, and LeF collected the references. XZ, HZ, and LiF analyzed the references. XZ and MX wrote the paper. All authors contributed to the design and interpretation of the study and to the writing of the drafts.

## Conflict of Interest

The authors declare that the research was conducted in the absence of any commercial or financial relationships that could be construed as a potential conflict of interest.
